# Association between pulse wave velocity from velocity-encoded MRI and advanced diastolic function indices assessed by speckle tracking strain analysis in Diabetes Mellitus type 1

**DOI:** 10.1186/1532-429X-13-S1-P63

**Published:** 2011-02-02

**Authors:** Linda D van Schinkel, Dominique Auger, Saskia G van Elderen, Nina Ajmone Marsan, Victoria Delgado, Arnold CT Ng, Jan W Smit, Jeroen J Bax, Albert de Roos, Jos J Westenberg

**Affiliations:** 1 LUMC, Leiden, Netherlands

## Introduction

Increased pulse wave velocity (PWV), a marker for aortic stiffness, has been linked to worse outcome such as heart failure. This potent marker can be accurately assessed with velocity-encoded MRI according to the transit-time method (Grotenhuis. JMRI 2009). Whether an increase in PWV translates in early diastolic dysfunction in diabetic patients remains unexplored.

## Purpose

Evaluate the association between PWV and diastolic function indices in patients with diabetes type1 (DM1).

## Methods

First normal values for age-related PWV were determined. Twenty-five healthy volunteers (age range 18-65years) were recruited and PWV-values of the total aorta were assessed with velocity-encoded MRI using a 1.5T MRI scanner (Philips, Best, the Netherlands). Linear regression between PWV and age defined normal values.

Next, PWV was assessed in 17 consecutive DM1 patients (mean age 48±8years). PWV was defined increased when the age-related value exceeds the normal value by 2×standard error (SE). Patients underwent subsequent 2D echocardiography for assessment of conventional diastolic function indices (isovolumetric relaxation time (IVRT), transmitral early (E) and atrial (A) peak filling velocities, E/A-ratio, E-deceleration time (DT)). Color-coded tissue Doppler imaging was applied to the 4-chamber view to determine mean mitral annulus velocity (E’). Furthermore, longitudinal speckle tracking strain analysis using apical 4-/2-/3-chambers views was performed to derive advanced diastolic indices [peak transmitral E-wave to strain rate during isovolumic relaxation (SRIVR)] and left atrium (LA) systolic strain. Subsequently, ratios of peak transmitral E-wave to mean E’ (E/E’) and of peak transmitral E-wave to strain rate during isovolumic relaxation (E/SRIVR) were calculated.

## Results

**Figure 1:**
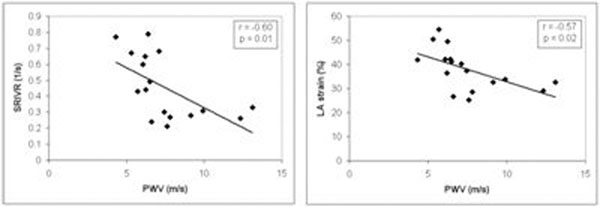
Association between PWV and SRIVR and LA strain.

Normal PWV age-relation was determined by linear regression in volunteer data: PWV=A×AGE+B, with A±SE=0.03±0.01m/s/year and B±SE=3.70±0.46m/s. In 17 DM1 patients, significant correlation was found between PWV and SRIVR and LA strain (Figure 1). A total of 7 patients showed increased age-related PWV. Mean PWV and echocardiographic diastolic function indices for patients with increased PWV (n=7) and for patients with normal PWV (n=10) are presented in Table1. Interestingly, conventional echocardiographic indices were similar between both patient groups whereas advanced diastolic function indices SRIVR, E/SRIVR and LA strain were significantly different (Table 1).

**Table 1:** MRI and echocardiographic results

	Normal PWV (N=10)	Increased PWV (N=7)	p-value
**PWV (m/s)**	6.0±0.8	9.6±2.3	0.006*
**IVRT (ms)**	76±10	84±30	0.52
**E/A (-)**	1.3±0.3	1.0±0.2	0.08
**DT (ms)**	214±50	235±51	0.43
**SRIVR (1/s)**	0.58±0.17	0.28±0.04	<0.001*
**E/E' (-)**	9±2	11±2	0.11
**E/SRIVR (cm)**	146±47	285±78	<0.001*
**LA strain (%)**	43±8	31±4	0.003*

## Conclusions

In patients with DM1, increased PWV is correlated with advanced diastolic function indices determined by speckle tracking strain analysis. Increased MRI PWV may translate in early left ventricular diastolic dysfunction.

